# Neurologic Evaluation of Premature Infants at Term Equivalent Age: Too Early or Too Late? A Scoping Review

**DOI:** 10.3390/medicina62061052

**Published:** 2026-05-28

**Authors:** Adrian Ioan Toma, Vlad Dima, Gabriela Corina Zaharie, Andreea Necula, Roxana Pavalache Stoiciu, Anca Roxana Bivoleanu

**Affiliations:** 1 Life Memorial Hospital, 010719 Bucharest, Romania; adrian.toma@prof.utm.ro (A.I.T.);; 2 Faculty of Medicine, University Titu Maiorescu, 040441 Bucharest, Romania; 3 Neonatology Department, Filantropia Clinical Hospital, 011132 Bucharest, Romania; 4 Department of Neonatology, Iuliu Hatieganu University of Medicine and Pharmacy, 400012 Cluj-Napoca, Romania; 5 Neonatal Intensive Care Unit, Clinical Hospital of Obstetrics and Gynaecology Cuza Vodă, 700038 Iași, Romania

**Keywords:** preterm infant, neurodevelopmental follow-up, term equivalent age, Amiel–Tison examination, general movements assessment, early intervention, cerebral palsy, motor optimality score

## Abstract

*Background and Objectives*: Early identification and referral for intervention of former premature infants at risk of neurodevelopmental impairment is considered a standard of care. The main purpose of this review was to assess the optimal timing of the first visit in a neurodevelopmental follow-up programme in order to identify at-risk infants in a timely and reliable manner. *Materials and Methods*: We considered three possible moments for the first evaluation: before 37 weeks postmenstrual age, at Term Equivalent Age (TEA, also known as 40 weeks postmenstrual age) and at 3–5 months corrected age (CA). A structured scoping review, informed by PRISMA-ScR principles, was performed. We searched PubMed/MEDLINE, Web of Science Core Collection, and Scopus from database inception through March 2026, combined with a Wohlin-type snowballing strategy. Two assessment techniques were evaluated: the Amiel–Tison neurological examination of the newborn and infant, and the General Movements Assessment (GMA). We collected data on sensitivity, specificity, and positive and negative predictive values at each of the three moments, and reviewed whether early intervention was associated with improved prognosis. *Results*: Intervention initiated before 12 months of age was associated with improved cognitive and motor outcomes in infancy compared with standard care; an additional benefit was observed when intervention started before discharge, particularly for cognitive outcomes in infancy. Both examinations showed very good specificity and negative predictive value at all three evaluation moments, consistent with their shared optimality concept. Sensitivity and specificity increased with the infant’s age. At each moment, the examinations identified (i) a high-risk group clearly requiring early intervention, (ii) a “grey zone” with uncertain evolution requiring closer surveillance, and (iii) a normal group with a very low risk of adverse outcomes. Combining two examination techniques at the same visit consistently improved discriminative and predictive performance. *Conclusions*: Evaluation at TEA alone may be too early because some abnormal findings normalize by 3 months CA, yet also too late for the most severely affected infants, who may manifest abnormal signs before term. We propose a stratified approach, with repeated evaluations using both the Amiel–Tison examination and GMA at 35–37 weeks postmenstrual age, at TEA, and at 3–5 months CA, in order to progressively identify infants at risk and refer them to appropriate early intervention. This proposal requires validation through prospective, well-designed research.

## 1. Introduction

The care of the high-risk premature infant does not end at discharge from the neonatal intensive care unit (NICU) [1]. NICU graduates should be enrolled in a structured developmental follow-up programme [2], the primary aim of which is the earliest possible detection of developmental problems [3]. Current guidelines for the early intervention of children at risk of cerebral palsy (CP) recommend immediate referral for diagnostic work-up once a patient is identified as at risk [4]. The identification of at-risk infants is based on a detailed history, standardized neurological examination, and neuroimaging [5].

Although the indication for neurodevelopmental follow-up is well established for former premature neonates [2], there is considerable variability in the structure and scheduling of follow-up programmes [6]. In a recent European survey, programme duration ranged from 2 to 5 years; the tests and examinations used differed across countries; and the date of the first visit ranged from term equivalent age (TEA) in parts of Italy to 3–6 months in most countries for which data are available to a single visit at 24 months in Germany [6]. In Romania (not included in that survey), according to the national guidelines revised in 2025, the follow-up programme lasts 2 years and the first assessment is scheduled at TEA.

Given the emphasis on early diagnosis and referral, it is tempting to identify infants at neurodevelopmental risk as early as possible. But how early? The central nervous system matures according to postmenstrual age [7], and classically the first relevant neurological examination in a former premature infant has been placed at TEA [7,8,9,10].

The main purpose of this review is to assess, based on the current state of knowledge, the optimal timing of the first follow-up visit—before TEA, at TEA, or at 3–5 months corrected age—in order to identify infants at risk in a timely and accurate manner and refer them for early intervention. A secondary aim is to examine whether an earlier diagnosis of risk of CP or neurodevelopmental impairment, resulting in an earlier referral to therapy, leads to a better outcome in former premature neonates.

## 2. Materials and Methods

### 2.1. Review Design

This work is a structured scoping review informed by the PRISMA Extension for Scoping Reviews (PRISMA-ScR) where applicable. We did not perform a full systematic review because the clinical questions were broad (comparative value of different assessment tools at different timepoints, and the effect of early intervention on outcome), and because the literature on each individual question has already been addressed by formal systematic reviews and meta-analyses, which are cited herein. This approach should lead to caution when interpreting the results of this review, which should be regarded more as a perspective on the authors’ experience and interpretation of the available literature. The completed PRISMA-ScR checklist is provided as Supplementary Material S1. A PRISMA 2020 flow diagram illustrating the search and selection process is provided as Supplementary Material S2.

### 2.2. Databases and Search Dates

Three databases were searched from their inception through 31 March 2026: PubMed/MEDLINE, Web of Science Core Collection, and Scopus. Reference lists of included systematic reviews and of the textbooks describing the two index examinations were additionally screened. No language restriction was applied, although only records with at least an English abstract were retained for full-text review.

### 2.3. Search Strategy

For each of the two index examinations, the following Boolean search blocks were used (adapted to the syntax of each database):

- (“Amiel-Tison” OR “Amiel Tison” OR “ATNAT”) AND (“preterm” OR “premature” OR “very low birth weight”) AND (“follow-up” OR “outcome” OR “neurodevelopmental”)
- (“General Movements” OR “GMA” OR “Prechtl” OR “fidgety movements” OR “cramped-synchronized” OR “motor optimality score”) AND (“preterm” OR “premature” OR “very low birth weight”) AND (“follow-up” OR “outcome” OR “cerebral palsy”)

**Figure 1:**
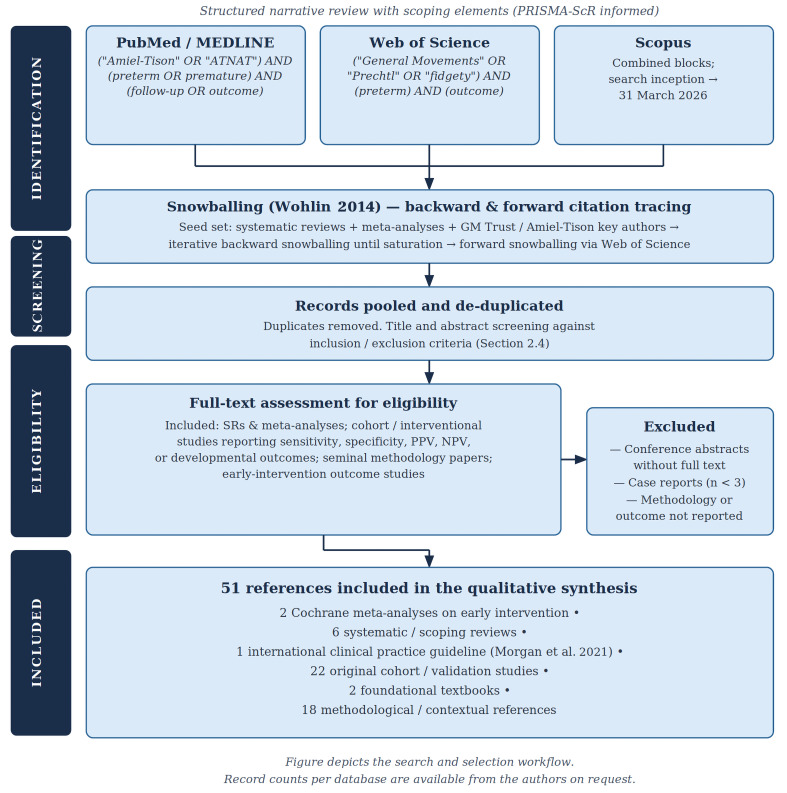
Search strategy and study selection workflow. Three databases (PubMed/MEDLINE, Web of Science Core Collection, Scopus) were queried in parallel and supplemented by a Wohlin-type snowballing procedure. Records were pooled, de-duplicated, and assessed for eligibility against the inclusion and exclusion criteria listed in Section 2.4. The diagram depicts the search workflow; record counts per database are available from the authors on request [4,12].

The database search was complemented by a snowballing strategy as described by Wohlin (2014) [11,12]: first a seed set of highly relevant papers was identified, followed by iterative backward snowballing (screening reference lists of included papers) until no new relevant records emerged, and then forward snowballing (screening papers that cited the seed set) through Web of Science and Google Scholar citation tracking. The overall workflow is depicted in Figure 1.

### 2.4. Inclusion and Exclusion Criteria

Inclusion criteria were as follows: (i) systematic reviews, meta-analyses, and clinical practice guidelines published in English; (ii) original observational or interventional studies describing the sensitivity, specificity, predictive values, or developmental outcomes associated with the Amiel-Tison examination and/or the General Movements Assessment in preterm infants; (iii) seminal methodological papers describing the examinations (including those authored by C. Amiel-Tison and colleagues, and by members of the General Movements Trust); and (iv) studies reporting outcomes of early intervention in infants at risk of CP. Exclusion criteria were as follows: (i) conference abstracts without a subsequent full-text publication; (ii) case reports with fewer than three participants; and (iii) papers that did not describe the examination methodology or the outcome assessment in sufficient detail. Priority was given to the most recent systematic reviews and meta-analyses, and to the most recent original studies when more than one study from the same group addressed the same question.

### 2.5. Limitations of the Search Strategy

We explicitly acknowledge that, despite the use of three databases and a structured snowballing procedure, this review does not meet the full methodological criteria of a systematic review. Selection bias is possible, particularly because the snowballing seed set was built around two well-defined assessment techniques and their authoring groups. Results and recommendations should therefore be interpreted with appropriate caution, and the clinical algorithm proposed at the end of this review should be regarded as hypothesis-generating rather than practice-changing.

### 2.6. Evidence Certainty: A GRADE-Informed Appraisal

Because a formal systematic review with meta-analysis was not performed, a standard GRADE (Grading of Recommendations, Assessment, Development and Evaluations) rating cannot be applied to individual outcomes in the conventional sense. Nevertheless, in the interest of transparency, we provide a GRADE-informed narrative appraisal of the certainty of evidence for each of the two principal questions addressed in this review. This approach follows the spirit of GRADE as originally proposed by Guyatt and colleagues [13], and as subsequently adapted for scoping and narrative reviews that synthesize heterogeneous bodies of literature.

Question 1—Predictive value of GMA and the Amiel-Tison examination at each timepoint. The evidence base consists primarily of prospective cohort studies and formal systematic reviews with meta-analyses [14,15,16]. In GRADE terminology, well-conducted prospective cohort studies addressing a diagnostic question begin at a “moderate” certainty level and may be upgraded if effect sizes are large, consistent, and reproducible across independent cohorts. For GMA at 3–5 months CA, the finding of absent fidgety movements is associated with very high sensitivity and specificity for CP across multiple independent cohorts and two systematic reviews [14,15]; accordingly, the certainty of evidence for this finding is assessed as MODERATE TO HIGH. For GMA at TEA, the predictive value of the persistent cramped-synchronized (CS) pattern is also supported by multiple studies [14,15,17,18], with some inconsistency in specificity attributable to the heterogeneous evolution of the poor-repertoire pattern; certainty is assessed as MODERATE. For the Amiel-Tison examination at TEA and at 3 months CA, the evidence is derived from smaller single-centre cohorts [19,20,21,22] and one large cohort study [22], with limited independent replication; certainty is assessed as LOW TO MODERATE. For examinations before 37 weeks, the evidence is more limited and largely derived from descriptive or single-centre studies [17,23,24]; certainty is assessed as LOW.

Question 2—Effect of early intervention on neurodevelopmental outcome. This question is addressed by two Cochrane systematic reviews with meta-analyses [25,26], representing the highest methodological standard available. However, the pooled certainty of the evidence is limited by the marked heterogeneity of included interventions, cohorts, and outcome measures—a limitation explicitly acknowledged by both meta-analyses [25,26]. In GRADE terminology, the certainty is rated as MODERATE for the effect of early intervention on cognitive outcomes in infancy and at pre-school age, LOW for motor outcomes beyond infancy, and LOW for the effect on CP incidence. The “LOW” ratings reflect the high heterogeneity (I^2^ frequently exceeding 75%) and the absence of sufficiently powered subgroup analyses isolating the contribution of early timing alone. These limitations do not invalidate the direction of the evidence, but they do limit confidence in the magnitude of the effect and in its generalisability.

A summary of this GRADE-informed appraisal is provided in Table 1.

### 2.7. Rationale for the Two Index Examinations

The Amiel-Tison examination and the General Movements Assessment were selected because (i) they are based on different conceptual frameworks—assessment of passive and active tone, reflexes, and synthesis for the Amiel-Tison examination [7,25] and observation of spontaneous movement patterns for GMA [8]—because (ii) both techniques can be applied, with methodological continuity, to premature infants before term, at TEA, and at 3–5 months post-term, and because (iii) both share the optimality concept of Prechtl [27]: a normal (optimal) result is a strong predictor of normal outcome, whereas a non-optimal result carries a less direct relationship to adverse outcome and therefore requires contextual interpretation. We did not include imaging modalities (cranial ultrasound, MRI) in this review; their complementary predictive value will be addressed in a separate work.

## 3. Impact of Early Identification and Intervention

Two systematic reviews with meta-analyses, published in 2015 and 2024 by the same Cochrane group, addressed the question of whether early intervention improves outcomes in preterm infants [25,26]. Early intervention was defined in both reviews as intervention occurring during the first 12 months of life, either in-hospital or after discharge [25,26]. The authors of both reviews emphasized that the heterogeneity of cohorts, studies, and interventions limited the strength of the pooled estimates [25,26]. The key findings of the two reviews, which provide the evidence base for Section 8 of this review, are summarized in Table 2.

Both meta-analyses examined cognitive and motor outcomes and the risk of cerebral palsy in preterm infants receiving early intervention versus standard follow-up. The earlier review [25] concluded that early post-discharge neurodevelopmental intervention programmes improved cognitive and motor outcomes in infancy and cognitive outcomes into pre-school age, but that the differences did not persist into adulthood. No significant difference in CP incidence was found between early and standard intervention groups [25]. The more recent 2024 meta-analysis confirmed an effect of early intervention on cognitive and motor outcomes in infancy, an improvement in cognitive outcomes at school age, no influence on motor outcomes beyond infancy, and no reduction in CP incidence [26].

In addition, the 2024 review included a subgroup analysis stratifying interventions by whether they began during the NICU stay or post-discharge [26]. No differential effect was observed for motor outcomes beyond infancy or for CP risk. For cognitive outcomes in infancy, both subgroups showed a positive effect; importantly, interventions initiated in-hospital showed less heterogeneity (I^2^ = 34%) than interventions started after discharge (I^2^ = 76%) [26]. For cognitive outcomes at pre-school and school age, the pre-discharge subgroup also showed a positive effect, although with fewer studies available. Interventions begun during the hospital stay had a greater impact on motor outcomes than interventions started after discharge [26].

Which interventions are considered effective? The EI SMART framework (Early Intervention in SensoriMotor, Attention and Regulation, Relationships, Therapist support) [28] proposes the promotion of self-initiated, developmentally appropriate motor activity, support of positive infant–parent interactions, support of self-regulation, and family involvement with attention to parental well-being. A systematic review of motor interventions in children with CP reached similar conclusions, emphasizing child-initiated movement, parental education, and environmental modification [29].

Indeed, many techniques are used in the case of early intervention programs for NICU graduates and discussing them in detail will need a separate systematic review. In addition, several reviews already discussed this topic and their results will be briefly summarized here. Regarding the interventions used in the case of patients at risk for CP, the evidence is low grade; no level I study could be identified, just level II–IV [29], with 10 types of intervention for level II–III studies and 8 types for level IV. Even if the interventions were different, the three components that were present in all the interventions and showed benefit were the following: (1) parent education—parents are trained in the provision of activities to progress the child in motor activities; (2) environmental modification—changing the surroundings of the child to elicit movements and increased motor behaviour; and (3) child-initiated movement—child initiates movements without guidance and encouragement of trial and error [29].

A review of preventive post-discharge intervention programmes for very preterm infants [30] included six programmes using different approaches, but all of them incorporated developmental support and support for the family. Interestingly, this review also discussed the person delivering the intervention: in four of the six studies, the intervention was delivered by a nurse, and in two studies by a physiotherapist [30].

The tests and examinations used as outcome measures were also very different between the studies (Bayley II, Alberta, Griffiths, Ages and Stages) [25,26,27,28,29,30], making comparisons difficult. The same heterogeneity in interventions and outcome measures has been noted by the two above-mentioned meta-analyses [25,26]. Considering this heterogeneity, a panel proposed a template for intervention description and replication (TIDieR) checklist and guide [31] to assist further studies in the field in order to increase homogeneity and applicability.

A paper from 2016 analysed the different types of early intervention programs and concluded that the greatest improvement in the motor pathways of preterm infants was obtained with interventions that focus on the parent–infant relationship and parent education [32]. Considering all of the above, in our opinion, more research is needed in order to establish the best interventions, evaluation methods, and outcome measures.

Two studies of interventions initiated during the NICU stay deserve mention. The first, performed on a very small cohort (*n* = 4) of patients with intraventricular haemorrhage who exhibited a cramped-synchronized (CS) General Movements pattern, applied Movement Imitation Therapy for Preterm Babies (MIT-PB): whenever a CS pattern was observed, the therapist and parent induced the corresponding normal movement pattern [33]. Despite the small sample, all four infants subsequently exhibited fidgety movements and had a normal developmental outcome at pre-school age [33]. The second study applied a structured neonatal physical therapy programme (positioning, active components including range-of-motion exercises, sensory and self-calming activities, multi-modal stimulation, and parent education) to a larger cohort of moderate-to-late preterm infants, with outcome assessment at 36.5 ± 1.7 weeks [34]. The authors concluded that this programme may improve aspects of neurobehaviour and the quality of General Movements in this population [34].

## 4. Predictive Value of Examinations Before TEA

Both the Amiel-Tison examination and the GMA were originally developed for use at term or shortly thereafter [7,8]. Nevertheless, both methods have been applied to premature infants before TEA, with specific adaptations.

### 4.1. Amiel-Tison Examination Before TEA

For the Amiel-Tison examination of the newborn, ten criteria have been established to assess normal neurological maturation of the premature infant: three items of passive tone (scarf sign, popliteal angle, return to flexion of the forearms), three items of active tone (pull-to-sit manoeuvre, pulling backward from the sitting position, righting reaction of the lower extremities and trunk), and four reflexes (finger grasp, response to traction, sucking reflex, crossed extension of the legs) [7,35]. Normative maturation for each item is tabulated for every week from 32 to 40 weeks of postmenstrual age [7]. In the premature infant, “non-alignment” of the results—three or more responses less mature than expected for the corrected age—is considered indicative of possible cerebral dysfunction; the authors of the method recommend repeating the examination one week later to confirm that the abnormal result is not transient [7]. A persistent abnormal result supports the diagnosis of cerebral dysfunction and referral for early intervention.

### 4.2. General Movements Assessment Before TEA

For GMA in the preterm period, normal and abnormal patterns are best characterized in relation to motor outcome. Two patterns at risk can be recognized: the early emergence of a cramped-synchronized (CS) pattern, and an atypical neonatal developmental trajectory. The CS pattern, first described in 2002, is a consistent, predominant movement pattern with strong predictive value for subsequent cerebral palsy [17]; impairment tends to be more severe when the CS pattern appears earlier in development [17]. A persistently normal movement pattern is consistently associated with normal motor outcome [18].

The interpretation of the third principal pattern—poor repertoire (PR)—was less clear in the original studies, as it could evolve either toward a normal pattern with fidgety movements or toward a pattern with absent fidgety movements [18]. A study published in 2020 addressed the importance of the developmental trajectory in these cases [23]: preterm infants were evaluated at approximately 31 + 1, 35 + 1, and 40 + 1 weeks. More than 90% of infants with a persistently normal or a PR-to-normal trajectory subsequently exhibited fidgety movements, while a persistent CS pattern or a PR-to-CS trajectory was associated with absent fidgety movements. A third group—persistent PR or normal-to-PR trajectory—was associated with abnormal fidgety movements [23]. Another study showed that in preterm infants born at less than 34 weeks, an examination near the time of discharge helps identify those requiring further assessment, with the strongest predictive performance for the later emergence of fidgety movements observed when GMA was performed at approximately 5 weeks post-term [24].

## 5. Predictive Value of Examinations at TEA

The theoretical rationale for both examinations is that neurological development of the preterm neonate proceeds according to postmenstrual age, and that the expected date of delivery (term equivalent age) provides a standardized moment for comparison [7,8,35]. Several studies have assessed the predictive value of these examinations at TEA for the neurological and developmental outcome of former premature neonates.

### 5.1. Amiel-Tison Neurological Assessment at Term (ATNAT)

Since the introduction of the method, ATNAT has consistently demonstrated good specificity and a good negative predictive value: a normal (optimal) examination is associated with normal outcome [36,37]. A 1988 study evaluated the predictive value of ATNAT alone and in combination with cranial ultrasound for neurodevelopmental prognosis at 12 months corrected age [38]. The combination of a favourable ultrasound and a normal neurological examination yielded a 98% probability of normal outcome at 12 months and a 100% probability of absence of major abnormality [38]. In the case of a normal neurological examination alone, the probability of a normal outcome at 12 months was 94% [38].

A 2005 study performed on infants with different risk factors for brain damage (both preterm and term) found good agreement between ATNAT and developmental follow-up results [19]. The correlation was strongest for optimal examinations followed by normal development: all children with an optimal ATNAT had normal follow-up results. In the severely non-optimal subgroup (*n* = 8), none had normal follow-up: 5/8 presented with severe deficits and 1/8 with moderate deficits [19]. The main limitation was the interpretation of the mildly non-optimal group: 14 of 18 patients in this group had normal development; in the moderate subgroup, results were distributed between normal, mild, moderate, and severe outcomes [19]. Agreement (Cohen’s kappa) was excellent for the subsequent neurological examination (κ = 0.83), and good for Bayley MDI (κ = 0.64) and PDI (κ = 0.74) [19].

A 2007 study assessed the predictive value of ATNAT for the developmental outcome at 20 months in a cohort of very low birth weight infants [20]. ATNAT showed a sensitivity of 0.61, specificity of 0.69, positive predictive value of 0.33, and negative predictive value of 0.88 for the prediction of motor outcome at 20 months CA [20]. The authors concluded that a positive prognosis of normal motor development can reliably be made from a neurological examination performed at TEA [20].

ATNAT has also been evaluated for the prediction of developmental problems in preterm infants without CP. In a cohort of preterm neonates (GA 29–37 weeks) with no cases of CP at follow-up, ATNAT was the only independent factor predictive of psychomotor and behavioural indices at 24 months CA, using a multivariate ANOVA model [21].

The largest study of the predictive value of ATNAT, published in 2013, enrolled 4030 preterm infants from the LIFT cohort, separated into training and validation sets [22]. A modified version of the examination was used, condensing several items into single-scored variables. A 16-item version was developed; in 13 items, an abnormal result was associated with suboptimal neurological examination at 2 years. In the training set, the presence of at least one abnormal item showed a specificity of 0.70, sensitivity of 0.48, positive likelihood ratio of 1.59, and negative likelihood ratio of 0.74. In the validation set, 12 of the 13 items remained associated with abnormal outcome at 2 years: sensitivity 0.64, specificity 0.56 overall; sensitivity 0.65, specificity 0.55 for the diagnosis of CP [22]. The authors concluded that a normal neurological assessment at term identifies a subgroup of preterm infants at lower risk of non-optimal neuromotor development at 2 years CA [22]. When gestational age was added to the model, the combination of GA > 33 weeks, normal imaging, and normal neurological examination at TEA was associated with normal outcome [22].

### 5.2. General Movements Assessment at TEA

Evidence for GMA at TEA comes from several cohort studies and systematic reviews. In one of the classical descriptions of the method [18], a pooled analysis reported a sensitivity of 94% and a specificity of 46–93%; the wide specificity range reflects the heterogeneous evolution of the poor repertoire pattern, which may either normalize or evolve toward absent fidgety movements. A persistent CS pattern at TEA is strongly predictive of later motor impairment [17,18]. A later onset and shorter duration of the CS pattern has been associated with later diplegia rather than tetraplegia [17], and segmental movements of the upper arms may further support this prediction [18]. A dyskinetic form of CP is suggested by finger spreading, a poor repertoire pattern, and circular movements of the fingers and arms [18].

A 2011 systematic review [14] found that the sensitivity of writhing movement quality for predicting adverse neurodevelopmental outcome was 75–100%, with 100% sensitivity for the detection of CP and a lower specificity of 40–48.2% (a single study in small-for-gestational-age infants reported higher specificities). For outcomes at 2–3 years, sensitivity was 55–100% and specificity 23–73% [14].

The strongest classical indication for GMA at TEA is the prediction of CP [8,18]. In a 2018 systematic review [15], the sensitivity for detecting CP from writhing-movement quality was 93% and specificity 59%, with positive predictive value 8–68% and negative predictive value 80–100% [15]. The authors suggest that the low specificity in this window reflects the substantial proportion of false-positive results, since many infants with abnormal writhing movements subsequently develop normal fidgety movements [15]. Again, the best predictive performance at TEA was achieved by a persistent CS pattern (sensitivity 70%, specificity 97%, positive predictive value 36–100%, negative predictive value 74–94%) [14].

Building on the three main GMA patterns at TEA (normal writhing, PR, CS), a score was developed to capture the other spontaneous movements visible during the writhing period—the General Movement Optimality Score (GMOS), and subsequently the revised GMOS-R [39,40]. The GMOS-R scores amplitude, speed, spatial range, proximal and distal rotations, and cramped character of upper- and lower-limb movements; normative percentile ranks are available for high-income and middle-to-low-income settings [40]. The incremental clinical value of GMOS/GMOS-R over pattern classification alone is not yet fully established; a large multicentre prospective cohort study in China has begun, one of whose aims is to correlate GMOS with the Motor Optimality Score-Revised at 3–5 months [41].

## 6. Predictive Value of Examinations at 3–5 Months Corrected Age

An important aspect to consider when suggesting such a protocol is the potential consequences of overdiagnosis, parental anxiety, and systemic costs. Regarding the risk of overdiagnosis, both examinations suggested here are based on the concept of optimality, which means they have great value in identifying normal patients. Good communication with families is essential: physicians should explain that infants in the grey zone are not necessarily abnormal, but that a repeated examination is needed. This limited-use algorithm for the early detection of CP is consistent with current guidelines for early detection and intervention [4,5]. One might argue that repeated examinations place a burden on families due to overdiagnosis and overuse of medical care. However, studies specifically evaluating unnecessary neonatal investigations did not identify the neonatal neurological examination as an unnecessary test placing burden on families, unlike, for example, MRI at TEA [42]. Regarding parental anxiety, studies have shown that parents prefer an early diagnosis [4,5], and that the main source of stress for families is not overdiagnosis or repeated controls, but the lack of information and the length of the waiting period before a diagnosis is established [43]. Our stratified approach aims to maintain continuous contact with families and to involve them in the early intervention process, which is the recommended approach for such programmes [44]. Regarding the economic implications, a 2025 analysis showed that early identification through universal screening of premature infants results in a lower cost per case, due mainly to a decrease in the cost of loss of well-being [45].

In a paper dedicated to the neurological examination at TEA, Amiel-Tison quoted the paediatric aphorism “Never trust a newborn” [36]. This caution is especially relevant for the neonatal neurological examination, because several factors modify the clinical picture between TEA and 3–5 months CA: maturation of the central nervous system of the young infant with normalization of some abnormal items, the delayed clinical expression of lesions that were asymptomatic due to immaturity, and—for GMA—the transition from one central pattern generator (CPG) to another, with the emergence of new movement patterns.

### 6.1. Amiel-Tison Examination at 3 Months CA

For the Amiel-Tison infant examination at 3 months CA, the study cited above [21] reported a sensitivity of 0.81, specificity of 0.57, positive predictive value of 0.38, and negative predictive value of 0.90. A normal neonatal AND 3-month neurological examination is strongly predictive of normal development; because of the transient neurodevelopmental dysfunction that resolves between TEA and 3 months, the authors recommended clinical follow-up at 3 months CA in all former premature infants, in order to increase the overall predictive value of the evaluation [21].

### 6.2. General Movements Assessment at 3–5 Months: Fidgety Movements

The 3–5-month window is characterized by the emergence of fidgety movements [8,18]. The sensitivity of GMA remains essentially unchanged, while specificity increases to 82–100%: a normal fidgety-movement pattern is strongly predictive of normal neurological outcome [18]. The absence of movements toward the midline and of foot-to-foot contact may be additional signs of risk for dyskinetic CP [18].

A 2008 systematic review [16] reported that the best predictor of neurodevelopmental outcome was the quality of GM during the fidgety period, with sensitivity > 92% and specificity > 82%.

The 2011 systematic review noted that abnormalities in the quality of fidgety movements at 12 weeks CA are more predictive of adverse outcome than abnormal writhing movements at TEA, with 92% sensitivity and 82% specificity for outcome at 12–24 months [14]. The same review showed 100% sensitivity for the detection of CP at 24 months, and lower sensitivities for detection of other developmental abnormalities (38–62.5%). For outcomes at 2–3 years, fidgety-movement quality showed a sensitivity of 54–100% and specificity of 46–100%, the lower values relating to psychomotor indices [14]. No association was found between abnormal fidgety movements and abnormal motor outcome in adolescence [14].

For the prediction of CP specifically, the absence of normal fidgety movements has the highest reported sensitivity (97%) and specificity (89%), with positive predictive value 8–100% and negative predictive value 80–100%—a better performance than GMA at TEA in comparative analyses that applied the Prechtl method and the Hadders-Algra method [15].

Analogous to the optimality concept behind GMOS, a Motor Optimality Score (MOS) has been developed for the fidgety period, later revised as MOS-R [46]. The initial study, published in 2019, showed that adding this score improved the prediction of motor outcome: 95% of infants in the study group who later developed CP did not exhibit fidgety movements, and 100% of them had a non-optimal MOS [46]. The score groups items into five domains—fidgety movements, observed movement patterns, observed postural patterns, movement character, and age-adequate motor repertoire—with an associated scoring guide [46]. The proposed clinical cutoffs of MOS-R are summarized in Table 3.

A scoping review concluded that a correlation exists between MOS and GMFCS level in children with CP—the lower the MOS, the higher the risk—with additional associations between specific domains (movement patterns, postural patterns, movement character) and outcome [47]. The authors noted, however, that MOS remains a relatively new tool, and that further research is needed to establish its reliability and validity [47]. A 2025 multinational reproducibility study concluded that MOS-R is highly reproducible, especially when used by experienced assessors [48]. A 2023 study investigating multiple evaluations between the preterm period and 3–5 months CA found that MOS-R alone was a statistically significant predictor of development at 1 year of age (OR 0.59; 95% CI 0.22–0.97; *p* < 0.02) [49].

## 7. Combining Two Examination Techniques at the Same Visit

An ideal evaluation tool for assessing a former premature infant should have both high discriminative and high predictive value [13,50]. In practice, no single tool meets all clinimetric criteria; authors have therefore recommended the use of more than one assessment at the same visit, in order to improve both discriminative and predictive performance [50]. The same recommendation emerged from a systematic review of the predictive value of spontaneous movements for the detection of CP risk [15].

Two studies have directly addressed this question of combining two techniques; their design and key findings are summarized in Table 4.

In the first study, Romeo and colleagues applied the GMA and the Hammersmith Infant Neurological Examination (HINE), both at 3 months CA, in a sample of 903 preterm infants [51]. A statistically significant correlation was found between the two methods (rs = 0.49; *p* < 0.001). When results were combined (HINE score and presence of fidgety movements), the correlation with the risk of CP was 0.89, higher than that of either examination alone (0.73 for GMA, 0.39 for HINE). The correlation was further strengthened when patients were stratified: in the group with HINE score < 50 and absent fidgety movements, all patients developed CP [51].

The second study, performed by our group, investigated the combined use of the Amiel-Tison neonatal and infant examinations and GMA for the prediction of CP, delayed sitting, and delayed/absent walking in a small cohort of preterm and term neonates (70 children, 62 preterm) [52]. A statistically significant correlation was observed between GMA and Amiel-Tison examinations at TEA (*p* < 0.001) and at 12 weeks CA (*p* < 0.001). To assess the cumulative predictive value, binary logistic regression models were built. No model combining the examinations at TEA reached statistical significance. At 12 weeks CA, two models reached statistical significance for the prediction of CP: (i) absent fidgety + abnormal scarf sign + abnormal popliteal angle, and (ii) absent fidgety + abnormal scarf sign + abnormal popliteal angle + abnormal axial tone + abnormal synthesis of the results. The same two models were significant for the prediction of delayed sitting [52]. Both models combine the global results of the two examinations with selected items of passive tone (scarf sign, popliteal angle) and axial active tone [52].

## 8. Proposed Stratified Algorithm for the First Evaluation Visits

The analysis of the data suggests three observations. First, both examinations have very good specificity and negative predictive value at all three evaluation moments—before TEA, at TEA, and at 3–5 months—as expected from their shared optimality framework [14,18,22] (see Table 5). Second, sensitivity and specificity both increase with advancing age, reflecting both the clarification of moderately abnormal Amiel-Tison findings [20,21,22] and the emergence of fidgety movements for GMA [18]. Third, at each evaluation moment, the examinations stratify patients into three categories: a clearly abnormal group (persistent CS pattern [17,18], severely non-optimal ATNAT [19,20], or MOS-R < 14 at 3 months [46]) for whom immediate referral to early intervention is indicated; a grey zone (persistent PR, moderately non-optimal ATNAT, or borderline MOS-R) requiring closer surveillance and repeat assessment; and a normal group with low risk of adverse outcome [14,15,18].

**Figure 2:**
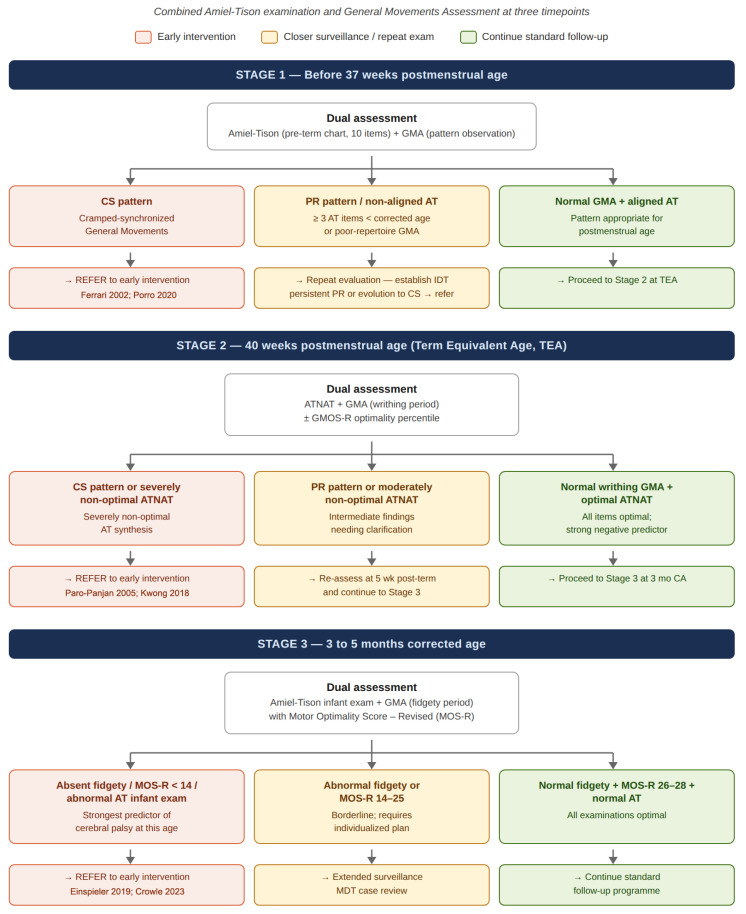
Proposed stratified algorithm for the first neurodevelopmental follow-up visits of former preterm neonates. At each of the three stages (before 37 weeks postmenstrual age; at term equivalent age; and at 3–5 months corrected age), a dual assessment combining the Amiel-Tison examination and General Movements Assessment stratifies infants into three outcome groups. Red boxes—immediate referral to early intervention; amber boxes—closer surveillance with repeat evaluation; green boxes—continuation in the standard follow-up programme. References in square brackets point to the primary studies supporting each decision node. AT, Amiel-Tison; ATNAT, Amiel-Tison Neurological Assessment at Term; CS, cramped-synchronized; GMA, General Movements Assessment; GMOS-R, General Movement Optimality Score—Revised; IDT, individual developmental trajectory; MDT, multidisciplinary team; MOS-R, Motor Optimality Score—Revised; PR, poor repertoire; TEA, term equivalent age [15,17,19,23,46,47].

No single moment is therefore “ideal” for the first examination. We propose a stratified approach with repeated evaluations using both techniques, in which patients are assigned to early intervention progressively as clinical signs become apparent (Figure 2). This approach is consistent with the observation that the more severe the impairment, the earlier the patient will become symptomatic [53]. Due to the absence of a formal qualitative assessment of the evidence, the proposal of an algorithm is thus problematic and it should be regarded with caution since there are no validation studies to support it. This represents more an opinion paper than an evidence-based synthesis. Obviously, more studies are needed in order to prove that this approach is correct and valid; it is not our aim to strongly recommend this approach as evidence-based. This represents more a concept and a basis for future studies.

Applied to our three-timepoint proposal, the algorithm operates as follows. (i) Before 37 weeks postmenstrual age, infants are evaluated by both techniques. A CS General Movements pattern prompts immediate referral to early intervention; a PR pattern prompts repeated assessments to establish an individual developmental trajectory—a persistent PR pattern or evolution to CS leads to referral. (ii) At TEA, patients not yet referred are re-evaluated. A CS pattern and/or a severely non-optimal ATNAT prompt referral; other patients are re-evaluated at 3 months CA, with an additional evaluation at 5 weeks post-term in cases of persistent PR. (iii) At 3 months CA, absent fidgety movements, a MOS-R < 14 or an abnormal Amiel-Tison infant examination leads to referral for early intervention. Patients with normal findings at all three timepoints continue in the standard follow-up programme.

## 9. Discussion

The present review was designed as a structured review with scoping elements, synthesizing, in the authors’ interpretation, the most relevant current evidence regarding the optimal timing of the first neurodevelopmental evaluation of former preterm infants. We used an organized search strategy combining three databases and a Wohlin-type snowballing procedure [11,12]; nonetheless, our approach is less rigorous than that of a formal systematic review, and the reader should interpret the synthesis accordingly. The most sensitive step in any snowballing approach is the composition of the seed set, and we prioritized systematic reviews, meta-analyses, and the most recent original studies in this set to mitigate this bias.

The target population for this review was the premature infants group without added congenital malformations and conditions, at risk for cerebral palsy and other neurodevelopmental problems. Besides this category of infants, there is also the group of premature infants with congenital malformations or syndromes. They are from the start a group at high risk that should be closely followed by a paediatric neurologist; they should from the beginning be included in an early intervention programme. A good example of this could be the case of patients with septo-optic dysplasia, who are usually phenotypically normal and will develop during the first year of life not only neuro-ophthalmological manifestations [54], but also developmental delay [55]. This condition is readily diagnosed by head ultrasound during the intra-uterine life or soon after (absence of the septum pellucidum)—after diagnosis, an MRI and ophthalmologic and hypophysis-function tests are needed in order to fully characterise the disease [55]; based on the above-mentioned risk, such patients should not be part of this algorithm, but should instead be referred for supplementary investigations and early intervention immediately.

Regarding early intervention, the two Cochrane meta-analyses [25,26] and their subgroup analyses (Table 2) indicate that the benefit on cognitive development is detectable in infancy and into pre-school age (with some effects extending to school age in the most recent review), and the benefit on motor development is largely confined to infancy. Neither review demonstrated a reduction in the incidence of CP. The absence of a preventive effect on CP is consistent with the biological definition of CP as a non-progressive disorder resulting from a static lesion, whose clinical manifestations may evolve over time but whose underlying neural injury is already present in the neonatal period [53,56]. The therapeutic aim in CP is not to cure the disease, but to prevent secondary complications and to limit functional consequences [56,57,58], even when early referral is considered the standard of care [4,5]. Given the heterogeneity of interventions and of outcome measures, these statements should be interpreted with caution, as emphasized by the authors of both meta-analyses [25,26].

Despite this heterogeneity, all the reviews and guidelines converge on common components of effective intervention: continuity of intervention, movement initiated by the child, and active parental education and engagement [4,28,29]. Guidelines recommend that intervention begin as soon as the risk of adverse development is suspected [4,5].

Concerning the central question of this review—whether the examination TEA is too early or too late to detect infants at risk—the answer is not categorical. Our findings can be summarized as follows:

- At each of the three moments considered (before TEA, at TEA, and at 3–5 months), both examinations show very good specificity and negative predictive value; a normal examination is consistently associated with a normal outcome. This is expected from the shared optimality concept of both techniques.
- Sensitivity and specificity increase with advancing infant age; nevertheless, at each moment, the examinations can identify high-risk groups that benefit from early intervention (Table 5).
- At each moment, the examinations define three categories: a high-risk group for whom early intervention is clearly indicated; a grey zone requiring closer surveillance; and a normal group whose outcome is likely to be normal.
- The combined use of two examination techniques increases discriminative and predictive value, particularly at 3 months CA [51,52] (Table 4).

One observational study of the timing of CP diagnosis showed that the more severe the impairment, the earlier signs appear and the sooner diagnosis is established [53]. A similar principle appears to apply to the early suspicion of developmental impairment in premature infants.

Before TEA, the presence and especially the persistence of a CS pattern are indicative of a high risk of motor impairment, especially CP, and should prompt referral to early intervention [17], as is probably the case for a persistent PR pattern [23]. This remains an area for further research, which is expected to clarify the grey-zone question—a robust multicentre protocol is currently in place [41]. Although the Amiel-Tison examination identifies abnormal patterns in the pre-term period [7], the supporting evidence is less extensive. Given the very good negative predictive value of the test at TEA [7,8,20], it is reasonable to infer that a normal alignment of maturation items in the preterm examination reflects a currently low risk. More studies are needed in this area to define the relationship between an abnormal Amiel-Tison examination before TEA and neuromotor outcome.

For examinations at TEA (both GMA and ATNAT), results are better standardized and more extensively studied. For ATNAT, the consistent finding across studies is that a normal examination at TEA is associated with normal outcome [19,20,21,22,36,37,38]. Adding other parameters—cranial ultrasound [38], stratification by GA < 33 weeks [22]—or combining extracted items with GMA findings [22,52] increases predictive capacity. A severely abnormal ATNAT is strongly associated with abnormal outcome [19], although the small sample size in the pivotal study argues for caution. The main limitation of ATNAT at TEA remains the interpretation of moderately non-optimal results, which may evolve toward either normal or abnormal outcomes and therefore demand closer surveillance.

For GMA at TEA, a high predictive value of the normal pattern for normal outcome is paralleled by a high-risk CS pattern [18,39,40], which should trigger referral to early intervention. The grey zone corresponds to the PR pattern, which may evolve either to normal or to absent fidgety movements at 3–5 months and requires closer surveillance [14,15,18]. GMOS-R may in the future refine the identification of high-risk infants within this pattern category, but further studies are required [39,40,41].

At 3–5 months CA, the predictive capacity of both examinations is highest, reflecting the clarification of moderately abnormal Amiel-Tison findings and the emergence of fidgety movements [14,18]. GMA at this age has the highest sensitivity, specificity, and negative predictive value for the risk of CP, with absent fidgety movements being the strongest predictor [14,15,18]. The MOS-R adds further predictive value for CP risk; the score strata summarized in Table 3 offer a transparent framework for triage at this age [46,47].

Taken together, these observations support the view that there is no single ideal age for the first evaluation. A stratified, multi-timepoint approach combining both techniques at 35–37 weeks, at TEA, and at 3–5 months CA (Figure 2) is consistent with the available evidence. This proposal is, however, hypothesis-generating: it must be validated in prospective studies with harmonized outcome measures.

### Strengths and Limitations

The main strength of this review lies in the structured search across three databases complemented by formal snowballing, and in the explicit focus on two examinations that share a methodological continuity from preterm age through 3–5 months. Limitations include the following: (i) the scoping rather than fully systematic design, with inherent selection bias despite the structured search [11,12]; (ii) the inclusion of one paper from our own group [52], which we have declared transparently; (iii) the exclusion of imaging modalities, whose additive predictive value will be addressed in a dedicated future review; (iv) the absence of a formal risk-of-bias assessment of the included primary studies; and (v) the absence of exact record counts per database, as records were pooled prior to de-duplication and individual database tallies were not retained. The completed PRISMA-ScR checklist and a PRISMA 2020 flow diagram are provided as Supplementary Materials S1 and S2, respectively.

## 10. Conclusions

Early intervention (before 12 months corrected age) in NICU graduates at risk of neurodevelopmental impairment has been shown to have a transient positive effect, particularly on cognitive outcome; more data are needed to characterize this effect more precisely. Initiating intervention before NICU discharge produces the same positive but transient effect on cognitive and motor outcomes in infancy. Effective interventions consistently share two features: child-initiated movement and a family-centred design that includes parental training and engagement.

Evaluation at TEA alone may be too early—some abnormal findings normalize by 3 months CA—and too late for the most severely affected infants, who may show signs before term. Although both GMA and the Amiel-Tison examination have their strongest predictive value at 3–5 months CA, the imperative of early identification favours a stratified approach with repeated evaluations between 35–37 weeks postmenstrual age and 3–5 months CA, progressively identifying infants at risk and referring them to appropriate early intervention. This proposal should be interpreted as a hypothesis-generating opinion paper rather than an evidence-based algorithm, and validated in well-designed, prospective research before being incorporated into general practice.

In our opinion, new aims for research in the future could be the following: *(i) to clarify the most efficient combinations of identification tools; (ii) to determine whether pre-TEA or TEA-based intervention alters long-term prognosis; (iii) to refine the clinical role of newer instruments such as GMOS-R and MOS-R; and (iv) to validate the proposed stratified algorithm in multicentre prospective cohorts*.

## Figures and Tables

**Table 1:** GRADE-informed appraisal of the certainty of evidence for the two principal questions addressed in this review.

Question/Outcome	Evidence Base	Main Limitations	GRADE Informed Certainty
GMA: absent fidgety movements predictive of CP (3–5 mo CA)	Multiple prospective cohorts; 2 systematic reviews [14,15]	Heterogeneous outcome definitions; variable follow-up duration	**MODERATE TO HIGH**
GMA: persistent CS pattern predictive of CP (preterm and TEA)	Multiple cohorts; systematic reviews [14,15,17,18]	Inconsistent specificity; PR pattern evolution unclear	**MODERATE**
Amiel-Tison examination at TEA and 3 months CA: predictive of adverse outcome	Smaller single-centre cohorts [19,20,21,22]; one large cohort [22]	Limited independent replication; variable outcome measures	**LOW TO** **MODERATE**
Any examination before 37 weeks: predictive of outcome	Descriptive and single-centre studies [17,23,24]	Small samples; no large prospective validation	**LOW**
Early intervention: effect on cognitive outcomes in infancy and pre-school age	Two Cochrane systematic reviews with meta-analyses [25,26]	High heterogeneity (I^2^ often >75%); variable interventions	**MODERATE**
Early intervention: effect on motor outcomes beyond infancy	Two Cochrane systematic reviews with meta-analyses [25,26]	Effect not sustained; high heterogeneity	**LOW**
Early intervention: reduction in CP incidence	Two Cochrane systematic reviews with meta-analyses [25,26]	No significant effect found; consistent across both reviews	**LOW**

CA, corrected age; CP, cerebral palsy; CS, cramped-synchronized; GMA, General Movements Assessment; TEA, term equivalent age. GRADE certainty levels adapted from Guyatt et al. [13]. A GRADE-informed (rather than fully formal GRADE) approach was applied because this is a scoping review and individual studies were not graded for risk of bias.

**Table 2:** Summary of findings from the two Cochrane meta-analyses on early developmental intervention programmes for preterm infants.

Outcome Domain	Spittle et al. 2015 [25]	Orton et al. 2024 [26]
**Cognitive outcome, infancy**	Improved vs. standard care	Improved vs. standard care
**Cognitive outcome, pre-school age**	Improved	Improved
**Cognitive outcome, school age**	Not significant	Improved
**Motor outcome, infancy**	Improved	Improved
**Motor outcome, beyond infancy**	Not persistent	No significant effect
**Incidence of cerebral palsy**	No significant effect	No significant effect
**Pre-discharge vs. post-discharge start (subgroup)**	Not stratified in detail	Cognitive infancy: both positive; in-hospital less heterogeneous (I^2^ 34% vs. 76%). Motor infancy: greater effect when started in-hospital.
**Heterogeneity of interventions**	High—limits pooled interpretation	High—limits pooled interpretation

CP, cerebral palsy. Data synthesized from Spittle et al. 2015 [25] and Orton et al. 2024 [26].

**Table 3:** Clinical interpretation of the Motor Optimality Score—Revised (MOS-R) at the fidgety-movement age. Cutoffs reported by Einspieler et al. [46] and the subsequent scoping review by Crowle et al. [47].

MOS-R Range	Clinical Interpretation	Suggested Action
**<8**	Associated with high-grade (GMFCS IV–V) cerebral palsy	Immediate referral; multidisciplinary rehabilitation planning
**<14**	High risk of cerebral palsy	Prompt referral for early intervention
**14–25**	Intermediate/borderline	Individualized assessment; extended surveillance
**26–28**	Considered normal	Continue standard follow-up programme

GMFCS, Gross Motor Function Classification System. Source data: Einspieler et al. 2019 [46]; Crowle et al. 2023 [47].

**Table 4:** Studies combining two examination techniques at the same follow-up visit.

Study	Cohort	Techniques Combined	Timepoint	Key Finding
**Romeo et al. 2008 [51]**	*n* = 903 preterm infants	GMA (fidgety) + HINE	3 months CA	Combined correlation with CP risk 0.89 vs. 0.73 (GMA) and 0.39 (HINE)
**Toma et al. 2026 [52]**	*n* = 70 (62 preterm, 8 term)	Amiel-Tison + GMA	TEA and 12 wk CA	Two binary logistic models at 12 wk CA significant for CP and delayed sitting

CA, corrected age; CP, cerebral palsy; GMA, General Movements Assessment; HINE, Hammersmith Infant Neurological Examination; TEA, term equivalent age.

**Table 5:** Reported predictive performance of ATNAT and GMA at the three evaluation timepoints considered in this review.

Timepoint	Examination	Outcome Assessed	Sens.	Spec.	PPV	NPV
**Before 37 wk**	GMA (CS pattern)	CP/motor impairment	—	—	—	—
**At TEA** **(40 wk)**	ATNAT [20]	Motor outcome at 20 mo CA (VLBW)	0.61	0.69	0.33	0.88
	ATNAT, 16-item modified [22]	Non-optimal neuromotor status at 2 yr (LIFT)	0.64	0.56	—	—
	ATNAT, 16-item modified [22]	CP at 2 yr (LIFT)	0.65	0.55	—	—
	GMA, writhing quality [14]	Adverse neurodev. outcome	75–100%	40–48.2%	—	—
	GMA, writhing quality [15]	CP	93%	59%	8–68%	80–100%
	GMA, persistent CS pattern [14]	CP	**70%**	**97%**	36–100%	74–94%
**3–5 mo CA**	Amiel-Tison infant exam [21]	Developmental performance at 2 yr	0.81	0.57	0.38	0.90
	GMA, fidgety quality [16]	Neurodev. outcome	>92%	>82%	—	—
	GMA, fidgety quality at 12 wk CA [14]	Adverse outcome 12–24 mo	92%	82%	—	—
	GMA, absent fidgety [15]	CP	**97%**	**89%**	8–100%	80–100%

ATNAT, Amiel-Tison Neurological Assessment at Term; CA, corrected age; CP, cerebral palsy; CS, cramped-synchronized; GMA, General Movements Assessment; NPV, negative predictive value; PPV, positive predictive value; Sens., sensitivity; Spec., specificity; TEA, term equivalent age; VLBW, very low birth weight. Values are reproduced from the original sources as cited. Em-dashes (—) indicate that the value was not reported in the cited source.

## Data Availability

No new data were created or analysed in this study. Data sharing is not applicable to this article.

## Supplementary Materials

The following supporting information can be downloaded at: https://www.mdpi.com/article/10.3390/medicina62061052/s1, Supplementary Material S1: PRISMA-ScR Checklist; Supplementary Material S2: PRISMA 2020 Flow Diagram. References [59,60] are cited in the supplementary materials.

## Abbreviations

AT	Amiel-Tison
ATNAT	Amiel-Tison Neurological Assessment at Term
CA	corrected age
CP	cerebral palsy
CPG	central pattern generator
CS	cramped-synchronized
GMA	General Movements Assessment
GM	General Movements
GMFCS	Gross Motor Function Classification System
GMOS-R	General Movement Optimality Score—Revised
IDT	individual developmental trajectory
MIT-PB	Movement Imitation Therapy—Preterm Babies
MOS-R	Motor Optimality Score—Revised
MRI	magnetic resonance imaging
NICU	neonatal intensive care unit
PR	poor repertoire
TEA	term equivalent age
