# Supplementary material for: Neurologic Evaluation of Premature Infants at Term Equivalent Age: Too Early or Too Late? A Scoping Review
Source: Medicina (Kaunas). 2026 May 28;62(6):1052. doi: 10.3390/medicina62061052 (PMC13304277; doi:10.3390/medicina62061052)
Supplement: Supplementary file 1 [file medicina-62-01052-s001.zip › Supplementary_S2_PRISMA_FlowDiagram.pdf]

Supplementary Material S2

PRISMA 2020 Flow Diagram for Scoping Reviews

Neurologic Evaluation of Premature Infants at Term Equivalent Age: Too Early or Too Late? A Scoping Review

Toma AI, Dima V, Zaharie GC, Necula A, Stoiciu R, Bivoleanu AR. Medicina 2025.

IDENTIFICATION

Records identified from: PubMed/MEDLINE (n = not retained separately)

Records identified from: Web of Science Core Collection (n = not retained separately)

Records identified from: Scopus (n = not retained separately)

▼

Records pooled and de-duplicated (n = approx. 1,100 records screened by title/abstract; exact counts unavailable — see limitation in Section 9.1)

Additional records from snowballing (Wohlin-type):

Backward snowballing from included systematic reviews and textbooks (n = not enumerated separately)

Forward snowballing via Web of Science and Google Scholar citation tracking (n = not enumerated separately)

▼

SCREENING

Records screened by title and abstract against inclusion/exclusion criteria (n = approx. 1,100+)

→ Records excluded at title/abstract stage: • Conference abstracts without full text • Case reports < 3 participants • Non-English without English abstract • No description of examination methodology or outcome assessment (n = majority excluded)

▼

Full-text records assessed for eligibility (n = approx. 80–100)

→ Full-text records excluded: • Insufficient methodological detail • Duplicate population from higher-priority source • Imaging-only studies (outside scope) • Intervention studies not addressing index examinations (n = approx. 30–40)

▼

INCLUDED

Sources of evidence included in scoping review and cited in final manuscript (n = 58 references)

Note: Record counts per individual database were not retained prior to pooling and de-duplication, as acknowledged in Section 9.1 of the manuscript. The approximate figures given above are estimates. Exact record counts are available from the corresponding author on request. This flow diagram adheres to PRISMA 2020 and PRISMA-ScR reporting guidance.

PRISMA 2020 reference: Page MJ, McKenzie JE, Bossuyt PM, Boutron I, Hoffmann TC, Mulrow CD, et al. The PRISMA 2020 statement: an updated guideline for reporting systematic reviews. BMJ 2021;372:n71. doi: 10.1136/bmj.n71.

PRISMA-ScR reference: Tricco AC, Lillie E, Zarin W, O'Brien KK, Colquhoun H, Levac D, et al. PRISMA Extension for Scoping Reviews (PRISMA-ScR): Checklist and Explanation. Ann Intern Med. 2018;169:467–473. doi: 10.7326/M18-0850.

Flow diagram template available at: https://www.prisma-statement.org/prisma-2020-flow-diagram
