# Supplementary material for: Neurologic Evaluation of Premature Infants at Term Equivalent Age: Too Early or Too Late? A Scoping Review
Source: Medicina (Kaunas). 2026 May 28;62(6):1052. doi: 10.3390/medicina62061052 (PMC13304277; doi:10.3390/medicina62061052)
Supplement: Supplementary file 1 [file medicina-62-01052-s001.zip › Supplementary_S1_PRISMA_ScR_Checklist.pdf]

Supplementary Material S1

PRISMA Extension for Scoping Reviews (PRISMA-ScR) Checklist

Neurologic Evaluation of Premature Infants at Term Equivalent Age: Too Early or Too Late? A Scoping Review

Toma AI, Dima V, Zaharie GC, Necula A, Stoiciu R, Bivoleanu AR. Medicina 2025.

SECTION / ITEM	ITEM #	PRISMA-ScR CHECKLIST ITEM	REPORTED ON PAGE / LOCATION
**TITLE**			
	**1**	Identify the report as a scoping review.	Title page; manuscript title
**ABSTRACT**			
	**2**	Provide a structured summary that includes (as applicable): background, objectives, eligibility criteria, sources of evidence, charting methods, results, and conclusions that relate to the review questions and objectives.	Abstract section
**INTRODUCTION — Rationale**			
	**3**	Describe the rationale for the review in the context of what is already known. Explain why the review questions/objectives lend themselves to a scoping review approach.	Section 1 (Introduction); Section 2.1 (Review Design)
**INTRODUCTION — Objectives**			
	**4**	Provide an explicit statement of the questions and objectives being addressed with reference to their key elements (e.g., population or participants, concepts, and context) or other relevant key elements used to conceptualize the review questions and/or objectives.	Section 1 (Introduction), final paragraph; Section 2.1
**METHODS — Protocol and registration**			
	**5**	Indicate whether a review protocol exists; state if and where it can be accessed (e.g., a Web address); and if available, provide registration information, including the registration number.	Section 2.1 — No formal protocol was pre-registered prior to the conduct of this scoping review. The review was conducted in accordance with PRISMA-ScR guidance (Tricco et al., 2018).
**METHODS — Eligibility criteria**			
	**6**	Specify characteristics of the sources of evidence used as eligibility criteria (e.g., years considered, language, and publication status), and provide a rationale.	Section 2.4 (Inclusion and Exclusion Criteria)
**METHODS — Information sources**			
	**7**	Describe all information sources in the search (e.g., databases with dates of coverage and contact with authors to identify additional sources), as well as the date the most recent search was executed.	Section 2.2 (Databases and Search Dates) — PubMed/MEDLINE, Web of Science Core Collection, Scopus; inception through 31 March 2026; supplemented by Wohlin-type snowballing (Section 2.3)
**METHODS — Search**			

SECTION / ITEM	ITEM #	PRISMA-ScR CHECKLIST ITEM	REPORTED ON PAGE / LOCATION
	**8**	Present the full electronic search strategy for at least 1 database, including any limits used, such that it could be repeated.	Section 2.3 (Search Strategy) — Full Boolean search strings presented for both index examinations
**METHODS — Selection of sources of evidence**			
	**9**	State the process for selecting sources of evidence (i.e., screening and eligibility) included in the scoping review.	Section 2.4 (Inclusion and Exclusion Criteria); Figure 1 (search workflow)
**METHODS — Data charting process**			
	**10**	Describe the methods of charting data from the included sources of evidence (e.g., calibrated forms or forms that have been tested by the team before their use, and whether data charting was done independently or in duplicate) and any processes for obtaining and confirming data from investigators.	Section 2.6 (Rationale for the Two Index Examinations) — Data were extracted and charted by the authoring team focusing on sensitivity, specificity, PPV and NPV at each of the three timepoints (Sections 4–6, Tables 2–4)
**METHODS — Data items**			
	**11**	List and define all variables for which data were sought and any assumptions and simplifications made.	Section 2 (Materials and Methods) — Variables: sensitivity, specificity, PPV, NPV for CP and other neurodevelopmental outcomes; effect of early intervention on cognitive and motor outcomes; three timepoints: <37 wk PMA, 40 wk PMA (TEA), 3–5 months CA
**METHODS — Critical appraisal of individual sources of evidence**			
	**12**	If done, provide a rationale for conducting a critical appraisal of included sources of evidence; describe the methods used and how this information was used in any data synthesis (if appropriate).	Section 2.5 (Limitations of the Search Strategy) — Formal risk-of-bias assessment was not performed; this is acknowledged as a limitation. Priority was given to systematic reviews, meta-analyses, and recent original studies.
**METHODS — Synthesis of results**			
	**13**	Describe the methods of handling and summarising the data that were charted.	Sections 3–7 — Narrative synthesis with tabular summaries (Tables 1–4); no quantitative pooling was performed
**RESULTS — Selection of sources of evidence**			
	**14**	Give numbers of sources of evidence screened, assessed for eligibility, and included in the review, with reasons for exclusions at each stage, ideally using a flow diagram.	Figure 1 (search workflow); Supplementary Material S2 (PRISMA 2020 flow diagram). Exact per-database record counts were not retained; records were pooled prior to de-duplication. 58 sources are cited in the final manuscript.
**RESULTS — Characteristics of sources of evidence**			
	**15**	For each source of evidence, present characteristics for which data were charted and provide the citations.	Sections 3–7; Tables 1–4; References 1–58
**RESULTS — Critical appraisal within sources of evidence**			
	**16**	If done, present data on critical appraisal of included sources of evidence (see item 12).	Not performed — acknowledged as limitation (Section 9.1)

SECTION / ITEM	ITEM #	PRISMA-ScR CHECKLIST ITEM	REPORTED ON PAGE / LOCATION
**RESULTS — Results of individual sources of evidence**			
	**17**	For each included source of evidence, present the relevant data that were charted that relate to the review questions and objectives.	Sections 3–7; Tables 1–4
**RESULTS — Synthesis of results**			
	**18**	Summarize and/or present the charting results as they relate to the review questions and objectives.	Section 9 (Discussion); Tables 1–4; Figure 2 (proposed algorithm)
**DISCUSSION — Summary of evidence**			
	**19**	Summarize the main results (including an overview of concepts, themes, and types of evidence available), link to the review questions and objectives, and consider the relevance to key groups.	Section 9 (Discussion); Section 10 (Conclusions)
**DISCUSSION — Limitations**			
	**20**	Discuss the limitations of the scoping review process.	Section 9.1 (Strengths and Limitations)
**DISCUSSION — Conclusions**			
	**21**	Provide a general interpretation of the results with respect to the review questions and objectives, as well as potential implications and/or next steps.	Section 10 (Conclusions); Section 9 (Discussion), final paragraphs
**FUNDING**			
	**22**	Describe sources of funding for the included sources of evidence, as well as sources of funding for the scoping review. Describe the role of the funders of the scoping review.	Funding section: 'This research received no external funding.'

Reference for PRISMA-ScR: Tricco AC, Lillie E, Zarin W, O'Brien KK, Colquhoun H, Levac D, et al. PRISMA Extension for Scoping Reviews (PRISMA-ScR): Checklist and Explanation. Ann Intern Med. 2018;169:467–473. doi: 10.7326/M18-0850.

PRISMA-ScR checklist available at: https://www.prisma-statement.org/scoping
